# Impact of Consultation with Registered Dietitians on Reducing Inappropriate Weight Gain in Pregnant Patients with Food Insecurity

**DOI:** 10.3390/nu17050789

**Published:** 2025-02-25

**Authors:** Kristen Lee Moriarty, Jacqueline Fleuriscar, Sarah Lindsay, Kelsey Manfredi, David O’Sullivan, Jessica Mullins

**Affiliations:** 1 Division of Maternal Fetal Medicine, Department of Obstetrics and Gynecology, UConn Health, Farmington, CT 06030, USA; 2 Department of Obstetrics and Gynecology, Hartford HealthCare, Hartford, CT 06102, USA; jfleuriscar24@gmail.com (J.F.); sarah.lindsay@hhchealth.org (S.L.); manfredi@uchc.edu (K.M.); jessica.mullins@hhchealth.org (J.M.); 3 Department of Research Administration, Hartford HealthCare, Hartford, CT 06102, USA; david.osullivan@hhchealth.org

**Keywords:** food insecurity in pregnancy, registered dietician counseling, inappropriate weight gain, interventions in food-insecure populations

## Abstract

**Background/Objectives**: Screening for food insecurity, while common practice in pediatric populations, remains novel in pregnancy. Food insecurity during pregnancy is associated with medical comorbidities that in turn confer additional obstetric risks to the maternal–fetal dyad. Few studies have evaluated the impact of interventions for patients with food insecurity in the prenatal period. This study first demonstrates the ease of FI screening in pregnancy using the Hunger Vital Sign™ and next assesses if providing patients with a referral to a registered dietician decreases the incidence of inappropriate weight gain in pregnant patients with food insecurity. **Methods**: A retrospective chart review was conducted from November 2019 to March of 2021 at a United States Northeast inner-city hospital-based clinic to identify patients with food insecurity in the prenatal period. All pregnant patients who screened positive for food insecurity were given an educational pamphlet with resources and offered a referral to a registered dietician. We compared the incidence of appropriate weight gain among these patients depending on whether they attended an appointment with a registered dietician. We defined appropriate weight gain following the recommendations of the Institute of Medicine (IOM) based on pre-pregnancy body mass index. Inferential statistics were performed to compare differences using univariate statistics, and multivariate regression was conducted to control for confounders, with an alpha of 0.05. **Results**: In total, 139 patients screened positive for food insecurity (FI); 52 (37.4%) attended an appointment with a registered dietician. Overall, 88 (61.9%) patients had inappropriate weight gain during pregnancy. Fewer patients who attended a visit with a registered dietician had inappropriate weight gain than those who did not attend a visit (27 [30.7%] vs. 61 [69.3%], *p* = 0.031, respectively). Both study groups’ demographics, comorbidities, and postpartum outcomes were comparable. **Conclusions**: We found that for pregnant individuals with food insecurity, consultation with a registered dietician was associated with a decrease in the incidence of inappropriate weight gain during pregnancy.

## 1. Introduction

Food security is defined as sufficient access to nutritious foods that meet an individual’s or a family’s dietary needs for a healthy lifestyle [1]. Conversely, food insecurity (FI) exists when individuals lack adequate access to fresh foods and often exists within households experiencing poverty [1]. According to the United States Department of Agriculture (USDA), 13.5% of American households experienced FI at some point in 2023. This equates to 18 million US households totaling 47.4 million people [2]. While FI screening is commonly performed in pediatric populations, it is not part of the standard obstetrical practice [3,4]. Moreover, prior research estimates higher rates of FI in vulnerable populations, such as pregnant patients. In a cohort of 451 patients undergoing care at the University of Cincinnati Medical Center, Sullivan et al. reported that at least one in five pregnant patients had FI [5]. Pregnant patients are a vulnerable population and are often faced with various socioeconomic stressors [6], such as FI, and this can impact maternal and fetal well-being. Prior research has demonstrated that pregnant patients welcome FI screening, citing they feel more supported by their clinician toward their mutual goal of a healthy pregnancy [7].

Previous research on patients experiencing FI has used an expanded screening tool called the United States Department of Agriculture’s (USDA) 18-question Household Food Security Scale. This tool may be challenging to adapt universally to practice due to its length, which makes it very challenging in prenatal care, where visits are often limited in time [1,8,9]. The American Academy of Pediatrics has implemented a validated, two-questionnaire screening tool (Hunger Vital Sign™) [10] that was initially successfully applied to the pediatric population [3]. This tool may be useful in a prenatal population, which shares many similarities with pediatric populations, such as being vulnerable, having a high sensitivity to medical interventions, and sharing ethical and legal considerations in medical practice. A prior integrative review by Pasha et al. included nineteen studies with reported household FI and pregnancy and noted several adverse physiological and psychological outcomes impacting pregnancy but reported several inconsistencies in the definitions and measures of FI, limiting conclusions [11]. This highlights the importance of defining a standardized and efficient method, such as the Hunger Vital Sign™, for screening patients who have FI, especially in prenatal populations.

FI has been associated with various health conditions, including obesity, hypertension, diabetes, and psychiatric concerns [1,12]. These health issues, when present during pregnancy, are subsequently risk factors for conditions including preeclampsia [13,14], poor fetal growth [15], perinatal mood disorders [16], and gestational diabetes [17], which in turn confer a risk of negative pregnancy outcomes [13,15,17].

There are limited studies that have evaluated adverse pregnancy outcomes in prenatal patients with FI and few studies have analyzed interventions for patients with FI and their impact on gestational weight gain, which is defined as the amount of weight gained during pregnancy [8,18]. A prior systematic review and meta-analysis by Nguyen et al. demonstrated that FI increased inadequate gestational weight gain (OR: 1.04; 95% confidence interval: 0.96, 1.13) [18]. Their study analyzed various maternal diets but the results were inconsistent with some links to maternal deficiency in vitamin E. Given the established link between individuals with food insecurity and adverse health outcomes, particularly in vulnerable populations such as pregnant individuals, it becomes critical to examine potential interventions [6,16,19].

This study aimed to assess differences in the incidence of inappropriate weight gain and associated outcomes among prenatal individuals with FI who attended a registered dietician consultation compared to those who did not. Following a quality improvement initiative project at this clinical site, all pregnant patients are screened for FI upon initiation of prenatal care using the Hunger Vital Signs screening questions. Appropriate gestational weight gain is based on guidelines published by the World Health Organization (WHO) based on the pre-pregnancy BMI with the recommended weight gain being stratified based on underweight, normal weight, overweight, and obese categories [20]. In this study, we define inappropriate weight gain as gaining too much or too little by the WHO recommendations for appropriate weight gain in pregnancy per BMI. Secondary outcomes were analyzed, including maternal and neonatal demographic and outcome data.

## 2. Materials and Methods

We completed a retrospective cohort study among patients with FI who received prenatal care at an inner-city hospital-based obstetrics clinic in the Northeast between 1 November 2019 and 1 March 2021, inclusive. Our study received approval from our healthcare system’s Institutional Review Board (IRB# E-HHC-2021-0057). All patient information was de-identified, and our research followed prevailing ethical principles. Inclusion criteria were patients who screened positive for food insecurity, started care on or after 1 November 2019, and delivered by 1 March 2021, inclusive, who initiated prenatal care in the first trimester in our clinic, and were aged 10 years old to 60 years old. We included patients with pre-existing diabetes or a history of gestational diabetes. We excluded patients who delivered before 30 weeks of gestation if they delivered at a different hospital than where prenatal care was obtained or if they were undelivered by 1 March 2021.

Following a quality improvement initiative project at this clinical site, all pregnant patients are screened for FI upon initiation of prenatal care using the Hunger Vital Signs screening questions (Online Resource 1) [3]. Patients are considered to have FI if they answer sometimes true or often true to either question. All patients who screened positive for FI were given an educational pamphlet. This pamphlet described community resources, including local food banks and pantries, and information about applying for the Supplemental Nutrition Assistance Program (SNAP) and the Special Supplemental Nutrition Program for Women, Infants, and Children (WIC). Patients with FI were also referred to an on-site registered dietician who discussed nutritional education and reviewed available community resources. During the consultation with the dietician, the patient’s diet history, anthropometrics, and estimated calorie needs for pregnancy were reviewed. The registered dietician (RD) discussed proper meal patterns and food safety and provided food resources such as SNAP and WIC. The RD referenced MyPlate and the recommended amount of servings per food group [21].

Our primary outcome was inappropriate weight gain among pregnant individuals with FI. We defined appropriate versus inappropriate weight gain in pregnancy as defined by the guidelines set by the Institute of Medicine (IOM) (Online Resource 2) based on pre-pregnancy body mass index (BMI) [5]. If patients gained less than or more than the recommended amount for their BMI at their initial prenatal visit, they were categorized as having inappropriate weight gain. We compared rates of inappropriate weight gain between those individuals who did and did not attend the registered dietician consult. We collected maternal demographic data, including maternal age at the date of delivery, ethnicity, marital status, weight at the first prenatal visit, BMI at the first prenatal visit, delivery weight, smoking status, substance use, parity at delivery, and ZIP code. We collected data on the following maternal comorbidities: pre-gestational and gestational diabetes, hypertensive disorders, and depression. We also evaluated postpartum visit attendance as a proxy for healthcare utilization and access. We collected the following infant data: APGAR scores at 1 and 5 min, neonatal intensive care unit admission, birth weight, and mode of delivery. These data were collected through a retrospective chart review. The process was standardized, and members of the research team were trained prior for appropriate data abstraction. The data were stored in a Redcap database.

We performed descriptive statistics for the study population. We summarized continuous, normally distributed demographic data using mean and standard deviation (SD) or non-normally distributed data with median and interquartile range (IQR). We summarized categorical data with frequencies and percentages.

We compared the incidence of inappropriate weight gain between those who attended versus those who did not attend a consult with the registered dietitian using a Pearson chi-square test. We used Student’s *t*-test and a Mann–Whitney U test to compare differences between continuous outcomes. We compared categorical data with a chi-square or Fisher’s exact test. We completed multivariate regression to control for demographic factors of ethnicity, age, ZIP code (resident of the city where the clinic is located or not), and substance use. We used an a priori alpha level of 0.05 for all analyses.

The sample size calculation for this study was based on a prior research article that reviewed weight gain in prenatal patients and from a prior quality improvement project we carried out at our current institution, in which we found that roughly 24% of patients were food-insecure. Of those patients, roughly ⅖ (43%) saw a nutritionist [5]. A power analysis was then performed to determine the number of participants required to power the study. A total sample size of 152, 38 in the group that saw a nutritionist and 114 in the group that did not, would afford 80% power to detect a difference between the group proportions of 0.237. We anticipated that the ratio of records would be approximately 1:3 (FI patients who did and did not see a nutritionist). Laraia et al. found that among food-insecure patients, 79% had inappropriate weight gain (either excessive or inadequate) [5]. The study was powered to detect a 30% relative difference in inappropriate weight gain, with FI patients who see a nutritionist having a lower incidence. A total sample size of 152, 38 in the group that saw a nutritionist and 114 in the group that did not, would afford 80% power to detect a difference between the group proportions of 0.237. The proportion in the nutritionist group is assumed to be 0.79 (i.e., 79% with inappropriate weight gain) under the null hypothesis and 0.553 (i.e., 30% lower) under the alternative hypothesis. The proportion in the group that did not see a nutritionist is 0.79. The test statistic used for this estimate is a two-sided Z test with pooled variance, using a significance level of 0.05.

## 3. Results

A total of 139 participants with FI met the inclusion criteria. Table 1 summarizes the study participants. The mean age of patients was 28.32 (standard deviation [2] of 6.33). The mean pre-pregnancy BMI was 29.16 kg/m2 (SD 7.58). Ninety (64.7%) patients identified as Hispanic. Fifty-two (37.4%) patients with FI attended a registered dietitian consultation. Of the 87 patients with FI who did not attend a consult, 15 (22%) were not referred; 88 (61.9%) patients had inappropriate weight gain, and of these patients, two-thirds had excessive weight gain.

Fewer participants who attended a registered dietician consultation had inappropriate weight gain compared to those who did not attend the consultation (27 (30.7%) vs. 61 (69.3%), *p* = 0.031, respectively (Table 2)). Some patients attended more than one visit with the registered dietician. In our secondary analysis, we found that at least one visit with the registered dietician was associated with a decrease in inappropriate weight gain.

We found no differences in demographics, baseline comorbidities, route of delivery, or neonatal outcomes between patients who attended a registered dietician consultation compared with those who did not (Table 3). We compared the attendance rates to the postpartum visit to assess the barriers to obtaining perinatal care. We found no difference between those who saw a registered dietician and those who did not and postpartum visit attendance (Table 3). We evaluated mode of delivery (vaginal delivery, vaginal birth after cesarean section [VBAC], and cesarean delivery) and found no difference between those who attended registered dietician consult and those who did not. We then excluded VBAC from the vaginal delivery category as there were few VBAC patients, and again, there was no difference in mode of delivery in comparing cesarean delivery to vaginal delivery.

## 4. Discussion

In 2009, the United States Department of Agriculture showed that 14.7% of all U.S. households were food-insecure [8]. The food insecurity rate increased during the COVID-19 pandemic, with 21.6% of the population identifying as food-insecure [20]. Identifying patients with food insecurity is imperative to improving clinical outcomes. Screening patients for food insecurity is relatively novel in maternal health. However, food insecurity has been studied in the pediatric population using the Hunger Vital Signs screening questions. This is a two-question tool adapted from the larger U.S. Household Food Security Survey Module [3].

Physiological changes during pregnancy are associated with increasing caloric requirements, thus requiring pregnant patients to have access to more foods. However, healthy foods often cost more per calorie. Access to suitable food markets is limited in lower-income regions. The COVID-19 pandemic created a further financial burden to access these healthy foods. Thus, screening for food insecurity is important for holistic care, especially in prenatal patients.

Prior research suggests that individuals with food insecurity during pregnancy are associated with adverse outcomes, such as anemia, gestational diabetes, depression, pre-eclampsia, and abnormal maternal weight gain [22]. Laraia et al. found that pregnant patients with food insecurity were associated with higher gestational weight gain and pre-gravid obesity [9]. However, few studies have evaluated the impact of interventions for patients with food insecurity in the prenatal period. A systematic review by McKay et al. described eleven studies performed in seven countries looking at interventions for patients with food insecurity during pregnancy [23]. Three studies were performed in the United States and the remaining studies were conducted internationally. Of these studies, only one listed an intervention involving food supplementation by increasing prenatal patients’ participation in WIC programs. Another international study by Frongillo et al. (2019) described implementing an antenatal intervention to increase nutritional knowledge during pregnancy. They found that this intervention reduced FI [8].

To date, most interventions for patients with food insecurity have focused on increasing community resources and participation. This is the first study to our knowledge that has provided prenatal patients with a referral to a registered dietician as an intervention to improve outcomes.

We note some limitations in our study. First, we did not include race in our analysis as race was inconsistently documented for all patients in our electronic health record during the study period. Additionally, our clinic has an established in-house registered dietician available to all patients. Therefore, our results may not be generalizable to other clinical settings without such resources, as our clinic was in an inner-city hospital setting, which may not be comparable to other communities. Additional studies are required in settings without this resource to better assess the generalizability of these results. Furthermore, 22% of patients with FI were not referred for consultation with a registered dietician. This lack of referral may have been attributed to the new referral process or patients declining services, and this was not always clearly documented. Another limitation includes the retrospective design and dependence on a limited time frame. This could restrict the capacity to make conclusive determinations regarding the long-term impacts of food insecurity and dietary consultations on maternal health outcomes. Finally, although this study gathers diverse demographic and clinical information, there may be additional factors that are not addressed in the present study that may be confounding the results, such as socioeconomic status, healthcare access, and existing social support systems. This may distort the interpretation of the findings in the present study. Our study found no associated changes in maternal or neonatal outcomes, but it was not powered to detect these differences.

## 5. Conclusions

Identifying patients who screen positive for food insecurity (FI) is crucial in providing comprehensive medical care. The reported incidence of FI in pregnant patients was estimated in 2020 to be 13.5%, yet screening for FI in pregnancy is not the standard of care [1]. FI in pregnancy is linked to adverse maternal and fetal complications, such as inappropriate weight gain and gestational diabetes [6,9,11,15,17]. While FI screening has become common practice in pediatric populations, screening for FI in prenatal populations has not been standardized. Various screening tools exist, with some much more cumbersome than others, thereby limiting their widespread implementation [11]. This study demonstrates the relative ease of FI screening in prenatal populations using the two-questionnaire screening tool developed by the American Academy of Pediatrics, the Hunger Vital Sign™ [3].

Screening for FI is integral to identifying individuals who may benefit from resources. Moreover, sharing access to such resources or possible interventions is of the utmost priority to improve health outcomes. In our study, registered dietician consultation in pregnant patients with food insecurity was associated with a decrease in the incidence of inappropriate weight gain during pregnancy. One registered dietician consultation was sufficient to provide this benefit.

Future research should investigate interventions targeting food insecurity for pregnant patients who screen positive for food insecurity to increase access to fresh and healthy food. There are improvements at multiple levels of the process to gain support for pregnant patients, including improving screening and then also adding various interventions, such as access to counseling. First, the screening process utilizes the Hunger Vital Signs screening questionnaire, which comprises two questions. This makes screening quick and efficient, but it may be prudent to expand this to include questions that appreciate the nuances of social determinants of health to capture those patients who may screen negative due to the narrow scope of a two-question questionnaire. In terms of the intervention, facilitating the scheduling of nutrition visits to occur immediately before or after their prenatal visit may increase the accessibility of these resources for patients as it integrates their care. Finally, following patients’ post-pregnancy and assessing if nutritional interventions during pregnancy improves overall dietary changes postpartum is important in assessing adherence.

## Figures and Tables

**Table 1:** Maternal and infant descriptive data for food-insecure population, N = 139.

Maternal Data		
Maternal Age at Delivery (years) (Mean ± STD)		28.32 ± 6.33
Gestational Age at Delivery (weeks, day) ^1^		38.50 ± 2.11
Gravity (Median, IQR)		3 (2–4)
Parity (Median, IQR)		1 (0–2)
Pre-pregnancy weight (lb)		164.17 ± 47.15
Pre-Pregnancy Body Mass Index (kg/m^2^) (Mean ± STD)		29.16 ± 7.58
Maternal Weight at Delivery (lbs) (Mean ± STD)		190.49 ± 45.67
Hispanic (N, %)		90 (64.7)
Non-Hispanic (N, %)		48 (34.5)
City Zip Code (N, %)		90 (64.7)
Suburban Zip Code (N, %)		49 (35.5)
Vaginal Delivery including Vaginal Birth After Cesarean (N, %)		89 (64.0)
Cesarean Section		50 (36.0)
Postpartum Visit Attendance	Yes	94 (67.6)
	No	45 (32.4)
Hypertensive Disease of Pregnancy		
Chronic Hypertension (N, %)	Yes	11 (7.9)
	No	128 (92.1)
Gestational Hypertension (N, %)	Yes	9 (6.5)
	No	130 (93.5)
Pre-eclampsia without severe features (N, %)	Yes	3 (2.2)
	No	136 (97.8)
Pre-eclampsia with severe features (N, %)	Yes	3 (2.2)
	No	136 (97.8)
Diabetes		
Type 1 Diabetes (N, %)	Yes	0 (0)
	No	139 (100)
Type 2 Diabetes (N, %)	Yes	6 (4.3)
	No	133 (95.7)
Gestational Diabetes Type 1 (N, %)	Yes	4 (2.9)
	No	135 (97.1)
Gestational Diabetes Type 2 (N, %)	Yes	10 (7.2)
	No	129 (92.8)
Drug Use, Mental Health		
Depression (N, %)	Yes	20 (14.4)
	No	119 (85.6)
Marijuana Use (N, %)	Yes	23 (16.5)
	No	116 (83.5)
Tobacco Use (N, %)	Yes	6 (4.3)
	No	133 (95.7)
Illicit Drug use (any other substance use) (N, %)	Yes	3 (2.2)
	No	136 (97.8)
Neonatal Data		
APGAR 1 min (Mean ± STD)		7.75 ± 1.24
APGAR 5 min (Mean ± STD)		8.85 ± 0.47
NICU Admission ^2^	Yes	14 (10.1)
	No	124 (89.2)

^1^ These are decimal days, not “x/7” days, so (e.g.) 37.6 = 37 w 6/10 days = 37w 4 d. ^2^ Missing 1 entry.

**Table 2:** Inappropriate weight gain in food-insecure patients.

			Registered Dietician Consult?	
			No	Yes
Inappropriate weight gain?	No	N	26	25
		% Inappropriate weight gain	51.0	49.0
		% Nutrition visit	29.9	48.1
	Yes	N	61	27
		% Inappropriate weight gain	69.3	30.7
		% Nutrition visit	70.1	51.9
	*p* = 0.031			

**Table 3:** Maternal and delivery data for food-insecure patients with and without registered dietician (RD) consult.

Demographic		Did Not Attend Consultation with RD ^n=87^	Attended Consultation with RD ^n=52^	*p* Value
Age in years		28.06 (6.5)	28.75 (6.08)	0.535 ^A^
Pre-pregnancy weight (lb)		167.65 ± 51.27	158.36 ± 39.10	0.263 ^A^
Pre-pregnancy BMI (kg/m^2^)		29.4	29.7	0.599 ^A^
Parity		1.55	1.37	0.586 ^B^
Gestational Age (weeks, days)		38.5	38.5	0.992 ^A,C^
Marital status (n, %)	Single	69 (79.3)	40 (76.9)	0.665 ^D^
	Married	17 (19.5)	12 (23.1)	
	Divorced	1 (1.1)	0 (0)	
Zip Code (n, %)	City	50 (35.5)	49 (35.3)	0.971 ^D^
	Suburb	91 (64.5)	90 (64.7)	
Postpartum Visit Attendance (n, %)	No	32 (36.8)	13 (25.0)	0.151 ^E^
	Yes	55 (63.2)	39 (75.0)	
Hypertensive disorders of Pregnancy				
Chronic Hypertension (n, %)	No	80 (92.0)	48 (92.3)	0.940 ^D^
	Yes	7 (8)	4 (7.7)	
Pre-eclampsia without severe features (n, %)	No	86 (98.9)	50 (96.2)	0.556 ^E^
	Yes	1 (1.1)	2 (3.8)	
Pre-eclampsia with severe features (n, %)	No	84 (96.6)	52 (100)	0.292 ^E^
	Yes	3 (3.4)	0 (0)	
Diabetes				
Type 1 Diabetes (n, %)	No	84 (96.6)	49 (94.2)	No stat
	Yes	3 (3.4)	3 (5.8)	
Type 2 Diabetes (n, %)	No	84 (96.6)	49 (94.2)	0.671 ^E^
	Yes	3 (3.4)	3 (5.8)	
Gestational Diabetes Mellitus Type 1 (n, %)	No	83 (95.4)	52 (100)	0.297 ^E^
	Yes	4 (4.6)	0 (0)	
Gestational Diabetes Mellitus Type 2 (n, %)	No	82 (94.3)	47 (90.4)	0.501 ^E^
	Yes	5 (5.7)	5 (9.6)	
Drug Use				
Tobacco (n, %)	No	84 (96.6)	49 (94.2)	0.671 ^E^
	Yes	3 (3.4)	3 (5.8)	
Marijuana (n, %)	No	74 (85.1)	42 (80.8)	0.510 ^D^
	Yes	13 (14.9)	10 (19.2)	
Illicit Drug use (n, %)	No	84 (96.6)	52 (100)	0.292 ^D^
	Yes	3 (3.4)	0 (0)	
Delivery Information				
Vaginal Delivery (including Vaginal birth after cesarean) (n, %)		55 (63.2)	31 (65.4)	0.996 ^D^
Cesarean Section (n, %)		32 (36.8)	18 (34.6)	0.996 ^D^
5 min APGAR (0–10) (Median, Interquartile range)		9 (7–10)	9 (6–9)	0.228
Infant Birth Weight (grams) (Mean ± SD)		3169 ± 540	3253 ± 608	0.402
NICU Admission (n, %)	No	78 (89.7)	46 (90.2)	0.919 ^D^
	Yes	9 (10.3)	5 (9.8)	

^A^ *t*-test; ^B^ Mann–Whitney U; ^C^ XXwYd converted to decimals, e.g., 38.5 = 38 w 3.5 d; ^D^ chi-square; ^E^ Fisher exact test. Values listed as mean (SD) or frequencies (%), except as indicated.

## Data Availability

Database cannot be made available on request due to institutional policies that forbid sharing of patient data.
